# Hepatitis C Virus-Induced Autophagy and Host Innate Immune Response

**DOI:** 10.3390/v9080224

**Published:** 2017-08-12

**Authors:** Stephanie T. Chan, Jing-hsiung James Ou

**Affiliations:** Department of Molecular Microbiology and Immunology, University of Southern California, Keck School of Medicine, 2011 Zonal Avenue, Los Angeles, CA 90033, USA; stephanietcchan@gmail.com

**Keywords:** hepatitis C virus, autophagy, innate immunity

## Abstract

Autophagy is a catabolic process that is important for maintaining cellular homeostasis. This pathway in hepatocytes is stimulated and controlled by the hepatitis C virus (HCV)—upon infection—to promote its own replication. HCV induces autophagy indirectly and directly through different mechanisms and temporally controls the autophagic flux. This enables the virus to maximize its replication and attenuate the innate immune responses that it activates. In this review, we discuss the relationship between HCV and autophagy, and the crosstalk between HCV-induced autophagy and host innate immune responses.

## 1. Introduction

Hepatitis C virus (HCV) is a positive single-stranded RNA virus that belongs to the *Hepacivirus* genus of the *Flaviviridae* family [1]. Its genome is 9.6 kb in length and encodes a polyprotein that is cleaved by host and viral proteases to yield ten mature viral proteins, which are the core protein, envelope proteins E1 and E2, the p7 ion channel protein, and nonstructural (NS) proteins NS2, NS3, NS4A, NS4B, NS5A and NS5B [2]. Most patients infected by HCV fail to clear this viral infection and remain asymptomatic for years before the development of severe liver diseases including cirrhosis and hepatocellular carcinoma. The ability of HCV to establish chronic infection in most of the patients that it infects is in part due to its regulation of critical signaling pathways in hepatocytes and its evasion of host innate immune responses.

**Figure 1:**
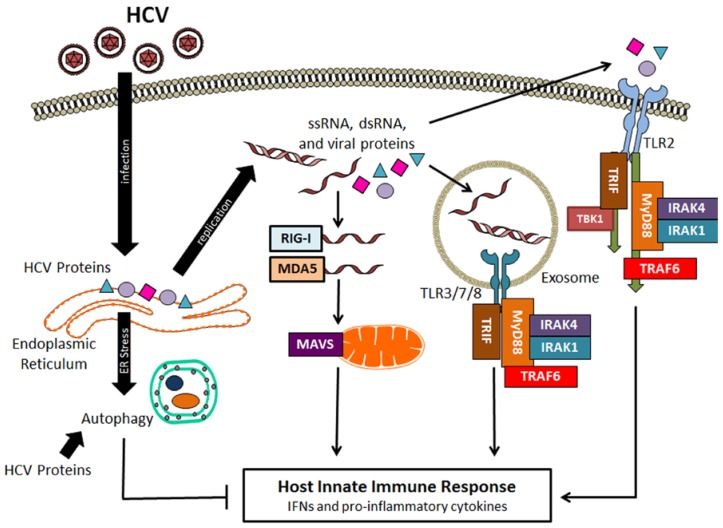
Hepatitis C virus (HCV) recognition by the host and HCV-induced autophagy. After infection, HCV genomic RNA and other HCV products such as double-stranded RNA (dsRNA) replication intermediates and viral proteins can activate retinoic acid-inducible gene I (RIG-I), melanoma differentiation-associated protein 5 (MDA5) and Toll-like receptors (TLRs) to activate signaling pathways to produce interferons (IFNs) and pro-inflammatory cytokines. HCV can also induce autophagy indirectly via the induction of endoplasmic reticulum (ER) stress or directly via its protein products to suppress host innate immune responses. IRAKs: Interleukin (IL)-1 receptor-associated kinases; MAVS: Mitochondrial antiviral signaling protein; MyD88: Myeloid differentiation primary-response protein 88; ssRNA: Single-stranded RNA; TRAF6: Tumor necrosis factor receptor-associated factor 6; TRIF: Toll/IL-1 receptor domain-containing adaptor protein inducing IFN-β.

Innate immunity is the host’s first-line antiviral defense mechanism, which responds immediately and non-specifically to viral infections [3,4,5]. A major innate immune response is the production of interferons (IFNs) and inflammatory cytokines. HCV infection activates a variety of pattern recognition receptors (PRRs) and triggers their downstream signaling pathways. PRRs, which include retinoic acid-inducible gene I (RIG-I) and Toll-like receptors (TLRs), recognize pathogen-associated molecular patterns (PAMPs). HCV is known to activate RIG-I, which recognizes the poly(U) motif in the 3’-untranslated region of the HCV genome. Upon its activation, RIG-I undergoes a conformational change that exposes its caspase recruitment domain (CARD) [6]. This CARD domain then binds to the mitochondrial antiviral signaling protein (MAVS, also known as VISA, IPS-1 or Cardif), which subsequently activates Tank-binding kinase-1 (TBK1) and IκB kinase ε (IKKε) to induce the phosphorylation and dimerization of interferon regulatory factor 3 (IRF3). The dimerized IRF3 is then translocated into the nucleus to induce the expression of IFNs. The activation of MAVS can also lead to the activation of nuclear factor-κB (NF-κB) to induce the expression of inflammatory cytokines [7]. Melanoma differentiation-associated protein 5 (MDA5)—another member of the RIG-I-like receptor family—can also be activated by HCV to trigger similar signaling pathways [8]. Other viral products from HCV replication can also activate host responses through TLRs (Figure 1). TLR3 in the endosome can recognize HCV double-stranded RNA (dsRNA), resulting in robust induction of chemokines and cytokines [9,10]. TLR2 senses HCV core and NS3 protein and activates inflammatory responses [11]. HCV infection can also trigger TLR7 and TLR8, which sense the HCV genomic RNA, to produce the pro-inflammatory cytokine tumor necrosis factor-α (TNF-α) [12]. TLR signaling involves myeloid differentiation primary-response protein 88 (MYD88), Toll/interleukin (IL)-1 receptor (TIR) domain-containing adaptor protein inducing IFN-β (TRIF), IL-1 receptor-associated kinases (IRAKs) and TNF receptor-associated factors (TRAFs), leading to the activation of NF-κB and IRFs [13] (Figure 1). As mentioned above, NF-κB and IRFs are transcription factors and once activated they will go into the nucleus where they will induce the expression of IFNs and pro-inflammatory cytokines. Protein kinase R (PKR), a dsRNA-dependent protein kinase, is another PRR that can be activated by HCV. The activated PKR will phosphorylate and inactivate the translation initiation factor eIF2α to suppress protein translation [14,15]. PKR recognizes the highly structured internal ribosome entry site (IRES) located near the 5’-end of the HCV genomic RNA [16,17]. Although earlier studies suggested that PKR could suppress HCV replication, subsequent studies indicate that PKR has no effect on HCV and that the inactivation of eIF2α by PKR is actually beneficial to HCV, as it suppresses the expression of IFNs and interferon-stimulated genes (ISGs) (for a review, see [18]). HCV NS5B, the viral RNA polymerase, had also been shown to use cellular RNA templates to synthesize dsRNAs to stimulate the expression of nucleotide-binding oligomerization domain-containing protein 1 (NOD1) and activate it. NOD1 is a nucleotide-binding oligomerization domain-like receptor or in short, NOD-like receptor (NLR). Its activation by HCV NS5B stimulates the expression of IFNs and pro-inflammatory cytokines [19]. 

Once IFNs are produced and secreted, they will activate IFN receptors to induce the expression of over 300 IFN-simulated genes (ISGs) to suppress viral replication. For example, the IFN-α/β receptor (IFNAR) activated by IFNs will recruit Janus kinase 1 (JAK1) and tyrosine kinase 2 (TYK2) to phosphorylate signal transducer and activator of transcription (STAT) 1 and 2. These STATs will dimerize to recruit IRF9 and form the ISGF3 complex, which will be translocated into the nucleus to activate genes that contain the IFN-stimulated response element (ISRE) [20]. Although HCV can trigger various signaling pathways to induce host innate immune responses, it has also developed mechanisms that inhibit these pathways. One well-documented mechanism is the cleavage of MAVS and TRIF by the HCV NS3 protease, which disrupts RIG-I and TLR3 signaling pathways [21,22,23]. HCV can also use autophagy to suppress innate immune responses (see Section 4 below). 

## 2. HCV and Autophagy

Autophagy (i.e., macroautophagy) is a catabolic process that is important for maintaining cellular homeostasis. It removes damaged organelles and protein aggregates from the cell and can also selectively remove intracellular microbial pathogens in a process known as xenophagy [24,25]. Xenophagy is a form of selective autophagy. It is initiated by the recognition of a molecular tag, often ubiquitin chains, on microbial pathogens. This recognition is mediated by a receptor such as the p62 sequestosome protein or the neighbor of breast cancer 1 (BRCA1) gene 1 protein (NBR1), which contains a microtubule-associated protein light chain 3 (LC3)-interacting region (LIR) and therefore can bind to LC3 to deliver the targets to autophagosomes [26]. LC3 is a cytosolic protein, but it is covalently linked to phosphatidylenthanolamine during autophagy. This lipidation of LC3 is important for its localization to autophagosomes and the formation of these membrane vesicles. Some viruses such as herpes simplex virus 1 (HSV-1) and Sindbis virus (SINV) are targets of xenophagy [27,28]. Autophagy can be activated by many stimuli including nutrient deprivation, oxidative stress, endoplasmic reticulum (ER) stress and microbial infections [29]. In the early stage of autophagy, a crescent membrane structure known as phagophore or isolation membrane is generated by different membrane sources in the cell. The phagophore membrane will subsequently expand and eventually form an enclosed double-membrane structure called the autophagosome. Autophagosomes mature by fusing with lysosomes to form autolysosomes, in which the cargos of autophagosomes are degraded by lysosomal enzymes for recycling [30]. Autophagy is a highly regulated process and its initiation is controlled by many factors such as the nutrient-sensitive kinases mammalian target of rapamycin (mTOR) complex 1 (mTORC1) and AMP-activated kinase (AMPK) [31]. A large body of evidence indicates that autophagy plays important roles in the regulation of innate immune responses to viral infections, including HCV.

HCV infection induces autophagy both in cell cultures and in the hepatocytes of chronically infected patients [32,33,34](for a review, see [35]). HCV may indirectly or directly induce autophagy. Indirectly, HCV can induce autophagy via the induction of ER stress (Figure 1). During infection—likely through the accumulation of its proteins in the ER—HCV causes the ER stress to activate PERK, activating transcription factor 6 (ATF6) and IRE1, which then further activate or induce the expression of downstream effectors in a process known as the unfolded protein response (UPR) [36,37,38,39,40]. The UPR alleviates the ER stress via multiple mechanisms. It induces the expression of chaperon proteins to facilitate protein folding and the proliferation of ER compartments to accommodate high protein load, attenuates protein synthesis, and initiates ER-associated degradation (ERAD) via proteasomes and autophagy to reduce the level of misfolded proteins [40]. Similarly, HCV may also indirectly induce autophagy via the induction of oxidative stress. It has been very well documented that HCV can induce oxidative stress via the induction of ER stress, the expression of nicotinamide adenine dinucleotide phosphate (NADPH) oxidases (Nox) 1 and 4, and the perturbation of mitochondrial functions (for reviews, see [41,42]). Recently, it was shown that the treatment of HCV-infected cells with antioxidants to alleviate oxidative stress would suppress HCV-induced autophagy, suggesting an important role for HCV-induced oxidative stress in the induction of autophagy [43]. Directly, HCV can induce autophagy by using its proteins to recruit or interact with autophagy proteins. The HCV p7 ion channel protein was found to bind to Beclin-1, a core component of the class III phosphatidylinositol-3-kinase (PI3KC3) complex that is important for the initiation of autophagy [44]. The HCV NS3/4A can bind to the mitochondria-associated, immunity-associated GTPase family M (IRGM) [45], which is a member of the interferon-inducible GTPase family and can interact with multiple autophagy-associated proteins including autophagy-related proteins (ATG) 5 and 10 to regulate autophagy. HCV infection has been shown to trigger IRGM-mediated phosphorylation of ULK1—an important initiation factor for autophagy—to induce autophagy [46]. Although IRGM also mediates the fragmentation of Golgi membranes—which become partially associated with autophagosomes—in HCV-infected cells [46], the biological significance of this Golgi fragmentation in the biogenesis of HCV-induced autophagosomes remains unclear and requires further study. HCV NS5B was found to interact with ATG5 and ATG12 in a yeast-two hybrid screen by two separate studies [45,47]. HCV NS4B can also induce the lipidation of LC3 and form complexes with Rab5, Vps34 and Beclin-1 [48,49]. Rab5 is a small GTPase important for membrane trafficking, and Vps34 is the catalytic subunit of PI3KC3. The overexpression of NS5A was also found to induce the expression of Beclin-1 [50]. It is likely that HCV combines both indirect and direct mechanisms to induce autophagy.

In addition to inducing autophagy, HCV also temporally regulates the autophagic flux. HCV infection was found to induce the accumulation of autophagosomes during the early stages of infection—i.e., up to the peak of HCV RNA replication and prior to the maturation and release of mature viral particles—without increasing autophagic protein degradation, which was enhanced only in the later stages of infection [49]. Further studies indicated that this was due to the induction of RUN Domain Beclin-1-interacting and cysteine-rich domain-containing protein (RUBICON) by HCV, which suppressed the fusion of autophagosomes and lysosomes. It inhibits the fusion between autophagosomes and lysosomes by sequestering UV radiation resistance associated protein (UVRAG) from the homotypic fusion and protein sorting (HOPS) complex [51,52,53,54]. This effect of RUBICON on the maturation of autophagosomes in HCV-infected cells was overcome in the later stage of infection due to the induction of UVRAG, which antagonized the effect of RUBICON [49]. The HCV subgenomic RNA replicon that expressed NS3–NS5B could also induce the expression of RUBICON and an incomplete autophagy, resembling the early stage of HCV infection [49]. Taguwa et al. [55] also reported a similar finding when they examined the HCV replicon cells, although they suggested that this incomplete autophagy might be due to the dislocation of vacuolar ATPase, which impaired the autolysosomal acidification. These studies indicated that HCV could temporally control the autophagic flux during infection, apparently to optimize its replication (see Section 3 below). 

## 3. Autophagy on HCV Replication

Autophagy plays a positive role in HCV replication, although theories on how it affects HCV replication are controversial. The HCV cycle can be divided into five major steps that include viral entry, protein translation, RNA replication, viral assembly and release [56]. Autophagy was shown to be required for HCV protein translation upon infection, but once HCV replication had been established, autophagy was dispensable [57]. Autophagy has also been shown to enhance HCV RNA replication by providing membrane sources for the assembly of HCV RNA replication complexes [58,59,60]. This use of autophagic membranes for its RNA replication would provide an explanation for why HCV delays the maturation of autophagosomes, as the premature fusion between autophagosomes and lysosomes would lead to the loss of HCV RNA replication complex that is associated with autophagosomal membranes. Autophagy has also been shown to play a role in HCV release, as the suppression of ATG7 and Beclin-1 expression or the inhibition of autophagy with chemicals reduced the release of HCV from cells [43,61,62]. It is possible that autophagy affects multiple stages of the HCV life cycle.

## 4. Suppression of Host Innate Immune Response by HCV-Induced Autophagy

**Figure 2:**
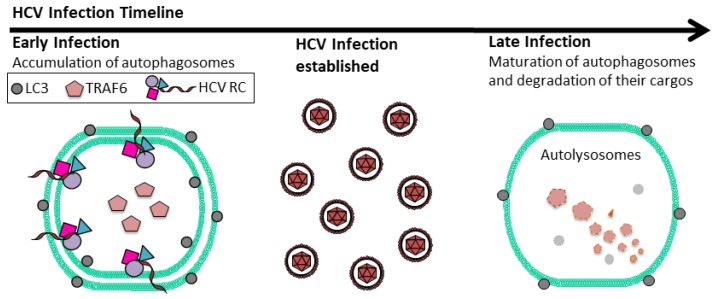
Temporal regulation of autophagic flux by HCV. In the early stage of HCV infection, autophagosomes accumulate in cells due to the delay of their maturation. This increases membrane areas for HCV RNA replication. TRAF6, an important adaptor protein of the host innate immune response, is also sequestered in autophagosomes. As HCV RNA replication climaxes, autophagosomes mature by fusing with lysosomes. At this time point, TRAF6 will undergo autophagic degradation. HCV RC: HCV RNA replication complex; LC3: Lipidated microtubule-associated proteins light chain 3.

HCV also uses autophagy to augment its replication by exploiting host innate immune responses. Shrivastava et al. showed that Beclin-1 or ATG7 knockdown in immortalized human hepatocytes enhanced the expression of type I IFNs and ISGs, suggesting a role for HCV-induced autophagy in the suppression of IFN signaling [63]. Ke and Chen [64] also found that ATG5 knockdown or the treatment of HCV-infected cells with chloroquine to suppress autophagy could enhance the expression of type I IFNs via RIG-I and activate the IFN signaling pathway. Their findings were consistent with a previous report, which demonstrated that mouse embryonic fibroblasts with ATG5 knockout—hence deficient in autophagy—were resistant to vesicular stomatitis virus (VSV) infection largely due to the hyperproduction of type I IFNs [65]. Recently, we also discovered that HCV-induced autophagy could deplete TRAF6, a member of the TNF receptor-associated factor that is important for mediating the signaling from members of the TNF receptor superfamily and TLRs (Figure 1). This depletion limited the production of host inflammatory cytokines. The depletion of TRAF6 by HCV was mediated by the p62 sequestosome protein, which recruited TRAF6 to autophagosomes for sequestration. The autophagic degradation of TRAF6 took place in the later stage of the HCV life cycle after HCV RNA replication had reached its peak, due to the delayed maturation of autophagosomes [66] (Figure 2). This finding further underscores the importance of temporal control of the autophagic flux by HCV, as it allows HCV to maximize the replication efficiency of its RNA and, in the meantime, sequester TRAF6 in autophagosomes for its eventual degradation to minimize host innate immune responses. In addition to the suppressive effect of HCV-induced autophagy on the expression of IFNs and inflammatory cytokines, Chandra et al. [67] also reported that HCV could suppress the antiviral effect of type I IFNs by suppressing the expression of IFNAR1—one of the two protein subunits of IFNAR—via autophagy, as they found that the inhibition of autophagy with chloroquine or via ATG7 knockdown could restore the IFNAR1 level in Huh7.5 cells infected by HCV and sensitize HCV to type I IFNs. The same group subsequently reported that the loss of IFNAR1 might be due to chaperone-mediated autophagy (CMA), as the treatment of HCV-infected cells with free fatty acid led to the loss of IFNAR1 via CMA [68].

## 5. Suppression of HCV Replication by IFNs via Autophagy

Although HCV could induce autophagy to suppress the IFN innate immune response, IFNs can also use autophagy to suppress HCV replication. By using transgenic mice that expressed the HCV NS3/4A protease in the liver, Desai et al. [69] studied the possible effect of NS3/4A—which cleaves MAVS— on the IFN response in vivo. To their surprise, they found that when these mice were challenged with VSV or a synthetic HCV genome they generated strong IFN-mediated responses, similar to what was observed in control mice. As the challenge of transgenic mice with the HCV genomic RNA led to the loss of HCV NS3, they examined the possible effect of IFNs on NS3 and discovered that IFN-β, but not IFN-α, could stimulate the autophagic degradation of HCV NS3/4A. Both IFN-α and IFN-β are type I IFNs. Although type I IFNs activate the same IFNAR, they are known to trigger different signaling responses due to the differences of their affinity to IFNAR1 and IFNAR2 and the stability of the ligand and receptor ternary complexes [70]. Kim et al. [71] also reported that IFN-β could induce SCOTIN—which bound to HCV NS5A and promoted its trafficking to autophagosomes for degradation—to suppress HCV replication. SCOTIN, also known as SHISA5, is a member of the SHISA family and a proapoptotic protein that is associated with the ER and nuclear membranes [72]. It is degraded by autophagy, although it does not affect the autophagic flux [71]. Kim et al. [71] found that the suppressive effect of over-expressed SCOTIN on HCV was dependent on autophagy, and if autophagy was inhibited then SCOTIN had no effect on HCV RNA replication. This observation is interesting but curious, as it is unclear why the binding of over-expressed SCOTIN to HCV NS5A did not suppress the formation of HCV RNA replication complex. Nevertheless, the studies of Desai et al. [69] and Kim et al. [71] indicated that IFN-β could target HCV proteins via different mechanisms for autophagic degradation to suppress HCV replication. Interestingly, IFN-λ1—a type III IFN that binds to IFNλ receptor 1 (IFNLR1)—was found to suppress the expression of ATG5 and γ-aminobutyric acid receptor-associated protein (GABARAP)—a protein related to LC3 and important for the formation of autophagosomes—to inhibit autophagy and HCV replication [73]. Thus, while IFN-β uses autophagy to degrade HCV proteins to suppress HCV replication, IFN-λ1 inhibits autophagy to suppress HCV replication.

## 6. Conclusions and Perspectives

Since the initial reports that HCV could induce autophagy in hepatocytes in 2008 [32,34], large amounts of information have been generated that advance our understanding of the relationship between HCV and autophagy. Although exactly how autophagy affects HCV replication remains a matter of controversy, there is general agreement that the autophagy induced by HCV plays a positive role in HCV replication. One mechanism by which autophagy promotes HCV replication is through the suppression of host innate immune responses. HCV uses autophagy to suppress IFN signaling. It also temporally controls the autophagic flux to sequester and deplete TRAF6 to maximize its RNA replication and its control of host innate immune responses. However, the host innate immune responses—including IFN-β and IFN-λ1—can also regulate the autophagic pathway to control HCV replication. Clearly, there is an interesting interplay between autophagy and IFNs in HCV-infected cells. While autophagy can negatively regulate the IFN response to enhance HCV replication, IFNs can also regulate autophagy, both positively and negatively, to suppress HCV replication.
